# Supplementary figures and images for: Proximal Binaural Sound Can Induce Subjective Frisson
Source: Front Psychol. 2020 Mar 3;11:316. doi: 10.3389/fpsyg.2020.00316 (PMC7062710; doi:10.3389/fpsyg.2020.00316)

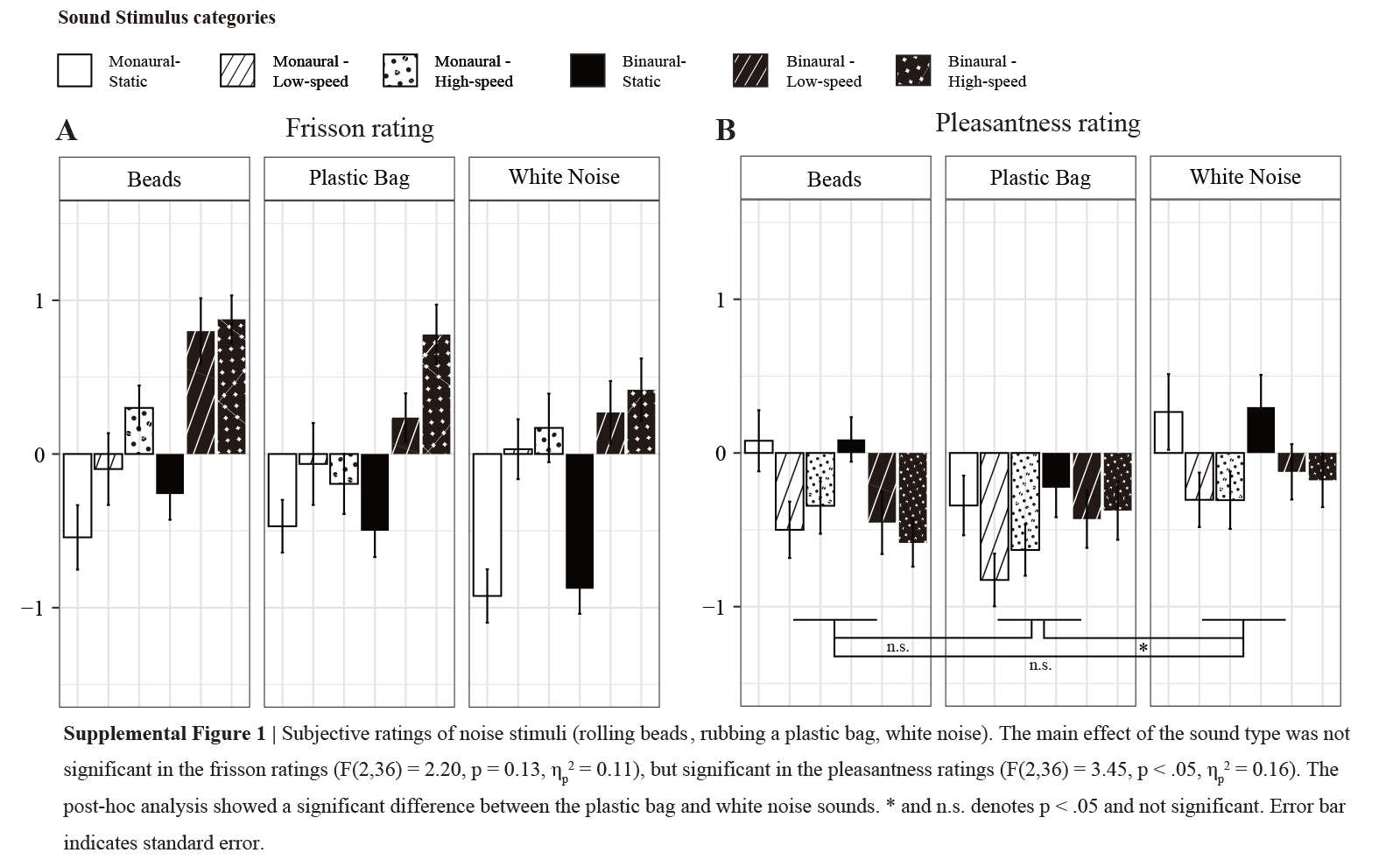

Supplement: Supplemental Figure 1 — Subjective ratings of noise stimuli (rolling beads, rubbing a plastic bag, white noise). (A) The main effect of the sound type was not significant in the frisson ratings [F(2, 36) = 2.20, p = 0.13, ηp2 = 0.11], but (B) significant in the pleasantness ratings [F(2, 36) = 3.45, p < 0.05, ηp2 = 0.16]. The post-hoc analysis showed a significant difference between the plastic bag and white noise sounds. * and n.s. denotes p < 0.05 and not significant. Error bar indicates standard error. [file Image_1.TIF]

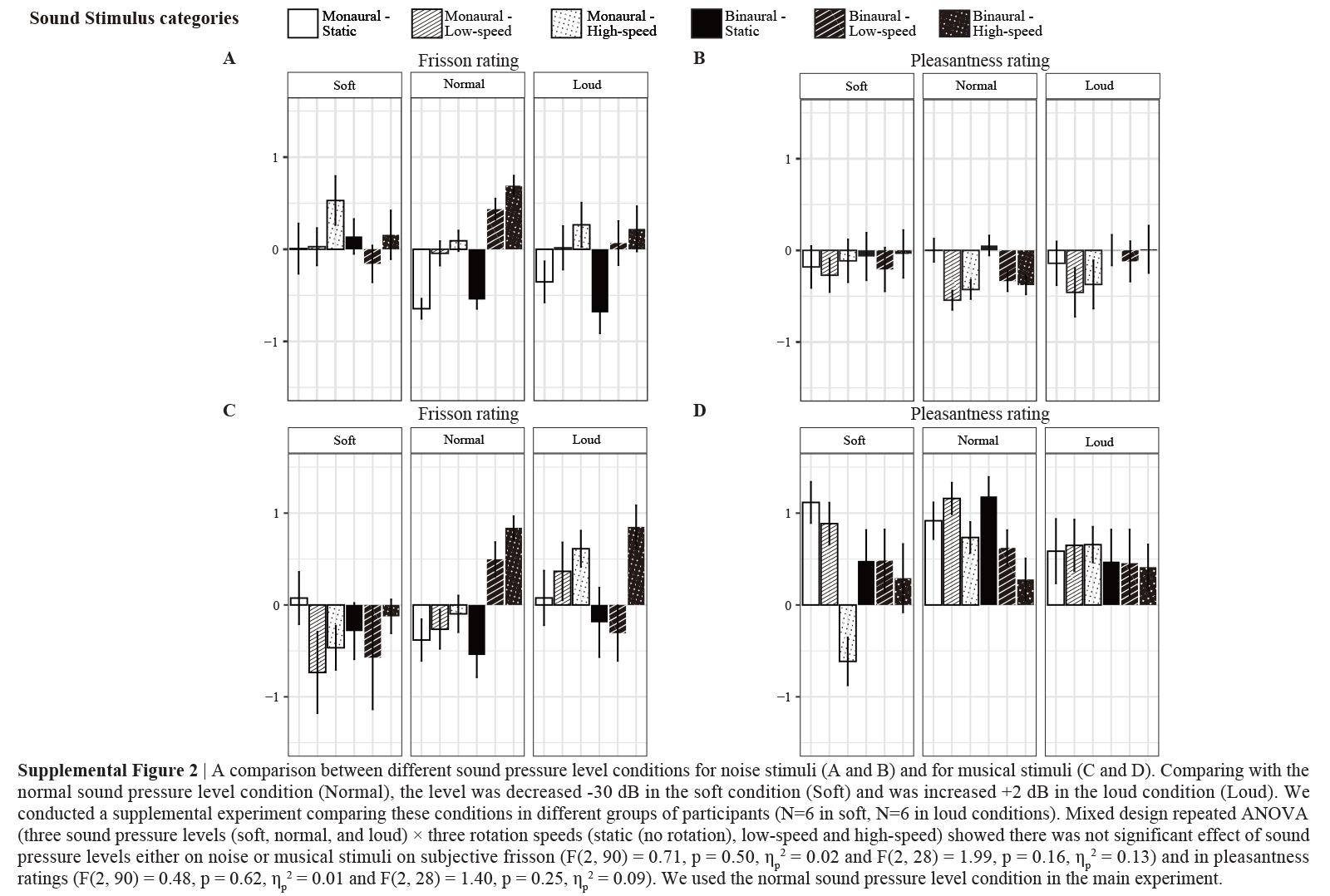

Supplement: Supplemental Figure 2 — A comparison between different sound pressure level conditions for noise stimuli (A,B) and for musical stimuli (C,D). Comparing with the normal sound pressure level condition (Normal), the level was decreased −30 dB in the soft condition (Soft) and was increased +2 dB in the loud condition (Loud). We conducted a supplemental experiment comparing these conditions in different groups of participants (N = 6 in soft, N = 6 in loud conditions). Mixed design repeated ANOVA [three sound pressure levels (soft, normal, and loud) × three rotation speeds (static (no rotation), low-speed and high-speed)] showed there was not significant effect of sound pressure levels either on noise or musical stimuli on subjective frisson [F(2, 90) = 0.71, p = 0.50, ηp2 = 0.02 and F(2, 28) = 1.99, p = 0.16, ηp2 = 0.13] and in pleasantness ratings [F(2, 90) = 0.48, p = 0.62, ηp2 = 0.01 and F(2, 28) = 1.40, p = 0.25, ηp2 = 0.09]. We used the normal sound pressure level condition in the main experiment. [file Image_2.TIF]

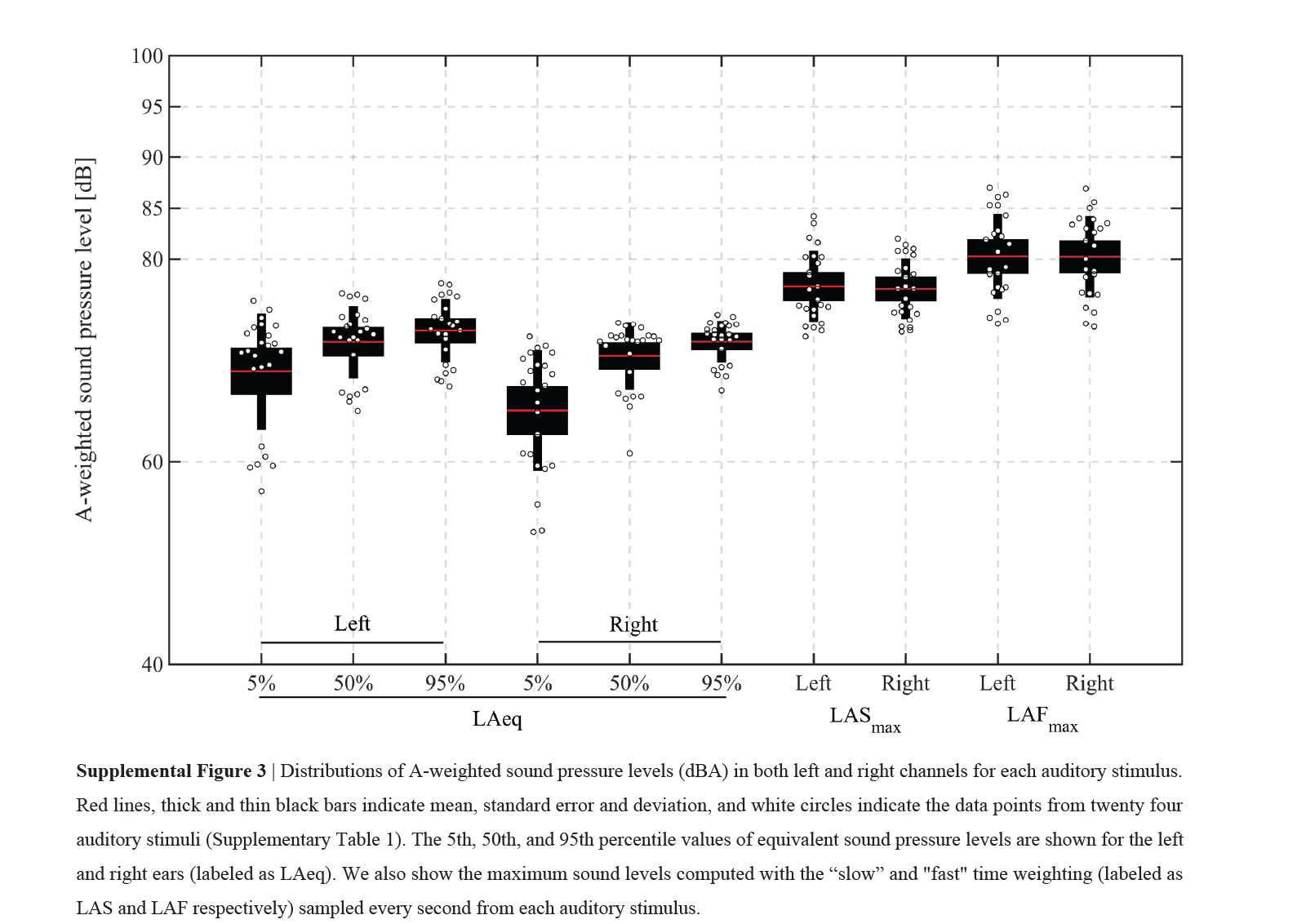

Supplement: Supplemental Figure 3 — Distributions of A-weighted sound pressure levels (dBA) in both left and right channels for each auditory stimulus. Red lines, thick and thin black bars indicate mean, standard error and deviation, and white circles indicate the data points from twenty four auditory stimuli (Supplementary Table 1). The 5th, 50th, and 95th percentile values of equivalent sound pressure levels are shown for the left and right ears (labeled as LAeq). We also show the maximum sound levels computed with the “slow” and “fast” time weighting (labeled as LAS and LAF, respectively) sampled every second from each auditory stimulus. [file Image_3.TIF]
