# Supplementary material for: Proximal Binaural Sound Can Induce Subjective Frisson
Source: Front Psychol. 2020 Mar 3;11:316. doi: 10.3389/fpsyg.2020.00316 (PMC7062710; doi:10.3389/fpsyg.2020.00316)
Supplement: Supplemental Table 1 — Distributions of A-weighted sound pressure levels sampled every second for each stimulus. The 5th, 50th, and 95th percentile values of equivalent sound pressure levels are shown for the left and right ears (labeled as LAeq). We also show the maximum levels computed with the “slow” and “fast” time weighting (labeled as LASmax and LAFmax respectively). We confirmed that all the auditory stimuli were below 85 dB for LAeq and LASmax, which is allowed noise level for 8 h exposure (US Department of Health and Human Services, 1998). For LAFmax, three auditory stimuli exceeded 85 dB but these were <90 dB, which is allowed noise level for 2.5 h exposure. [file Table_1.pdf]

SUPPLEMENTAL TABLE 1 | Distributions of A-weighted sound pressure levels sampled every second for each stimulus. The 5th, 50th, and 95th percentile values of equivalent sound pressure levels are shown for the left and right ears (labeled as LAeq). We also show the maximum levels computed with the “slow” and "fast" time weighting (labeled as LASmax and LAFmax respectively). We confirmed that all the auditory stimuli were below 85 dB for LAeq and LASmax, which is allowed noise level for 8 hours exposure (US Department of Health and Human Services. 1998). For LAFmax, three auditory stimuli exceeded 85 dB but these were less than 90 dB, which is allowed noise level for 2.5 hours exposure.

	Sound	Speed	LAeq						LASmax		LAFmax	
			Left			Right			Left	Right	Left	Right
			5	50	95	5	50	95				
Binaural	Beads	0	70.7	71.9	72.1	67.8	71.8	72.0	75.4	75.4	79.0	79.0
		Low	70.9	71.9	74.3	60.8	71.4	73.0	78.4	82.0	81.9	86.9
		High	69.1	70.5	71.0	60.7	68.8	71.1	77.0	79.1	82.5	85.0
	Plastic bag	0	70.4	72.8	73.0	70.1	72.4	72.6	75.0	74.8	77.0	76.7
		Low	72.6	74.3	76.0	62.7	72.2	73.7	80.2	81.4	82.2	83.4
		High	73.2	73.5	74.1	70.7	72.4	72.9	78.7	78.8	82.8	84.0
	White noise	0	69.3	72.2	72.6	65.8	72.0	72.5	73.2	73.2	73.6	73.6
		Low	73.5	74.5	76.5	64.9	71.9	73.4	80.3	80.8	81.5	83.0
		High	72.4	73.3	73.6	68.9	71.8	72.4	77.3	77.3	80.7	81.8
	Music	0	59.4	65.9	68.1	59.6	66.7	69.0	72.3	73.3	74.2	75.2
		Low	59.7	66.8	67.9	53.1	60.8	69.3	73.3	77.1	76.7	80.0
		High	60.5	65.0	67.4	53.2	65.4	67.0	75.1	74.7	78.6	78.2
Monaural	Beads	0	71.7	72.2	72.5	67.0	71.9	72.0	75.5	75.3	79.2	78.8
		Low	71.4	74.0	75.1	69.5	72.4	73.4	82.1	80.8	87.0	85.6
		High	71.6	72.8	73.8	67.5	70.6	71.9	79.6	78.2	85.3	83.9
	Plastic bag	0	70.8	73.1	73.4	69.4	72.3	72.5	75.3	74.6	77.2	76.5
		Low	73.4	76.6	77.6	70.7	73.7	74.5	84.2	81.0	86.1	83.0
		High	75.9	76.3	76.7	72.3	73.4	73.7	81.6	78.5	86.3	83.5
	White noise	0	69.5	72.5	72.9	68.6	71.9	72.3	73.6	72.9	74.0	73.3
		Low	74.2	76.5	77.5	71.2	73.5	74.3	83.5	80.4	85.3	82.6
		High	75.0	76.1	76.3	71.4	73.2	73.5	80.2	77.1	84.3	81.3
	Music	0	59.6	66.4	68.7	59.3	66.2	68.5	72.9	72.8	74.8	74.7
		Low	57.1	66.6	69.5	55.8	66.4	69.4	76.0	76.1	78.5	78.5
		High	61.5	67.1	69.0	59.6	66.4	68.4	74.4	74.0	77.2	76.6
