# Supplementary material for: Proximal Binaural Sound Can Induce Subjective Frisson
Source: Front Psychol. 2020 Mar 3;11:316. doi: 10.3389/fpsyg.2020.00316 (PMC7062710; doi:10.3389/fpsyg.2020.00316)
Supplement: Supplemental Table 2 — Results of correlation analysis between subjective frisson rating and psychological assessment (State-Trait Anxiety Inventory-JYZ: STAI and UPPS-P Impulsive Behavior Inventory in Japanese). A significant correlation was not found between these parameters. [file Table_2.pdf]

SUPPLEMENTAL TABLE 2 | Results of correlation analysis between subjective frisson rating and psychological assessment (State-Trait Anxiety Inventory-JYZ: STAI and UPPS-P Impulsive Behavior Inventory in Japanese). A significant correlation was not found between these parameters.

	Music stimuli		Noise Stimuli	
Personality trait measures	coefficient	p value	coefficient	p value
Premeditation (lack of)	0.09	0.7	0.24	0.31
Urgency	0.03	0.9	-0.08	0.74
Sensation Seeking	0.10	0.67	0.03	0.89
Perservance (lack of)	0.32	0.18	-0.15	0.55
STAI (State)	0.13	0.59	-0.35	0.14
STAI (Trait)	0.19	0.44	-0.28	0.25
