# Supplementary material for: Proximal Binaural Sound Can Induce Subjective Frisson
Source: Front Psychol. 2020 Mar 3;11:316. doi: 10.3389/fpsyg.2020.00316 (PMC7062710; doi:10.3389/fpsyg.2020.00316)
Supplement: Supplemental Table 3 — Results of correlation analysis between subjective rating (frisson/pleasantness) and acoustic parameters (Figure 3 shows scatter plots of these results) in the condition of non-musical sound (noise) stimuli. There were significant correlations between the variance of ILDs at specific frequency and the magnitude of subjective frisson rating. Loudness and sharpness were also significantly correlated with subjective frisson and pleasantness rating in several conditions (*, **, and *** denote p < 0.05, 0.01, and 0.001, respectively). Examples of sound stimuli used in this study. [file Table_3.pdf]

SUPPLEMENTAL TABLE 3 | Results of correlation analysis between subjective rating (frisson / pleasantness) and acoustic parameters (Figure 3 shows scatter plots of these results) in the condition of non-musical sound (noise) stimuli. There were significant correlations between the variance of ILDs at specific frequency and the magnitude of subjective frisson rating. Loudness and sharpness were also significantly correlated with subjective frisson and pleasantness rating in several conditions (*, **, and *** denote $p < 0.05$, 0.01, and 0.001, respectively).

Sensory evaluation	[sound types]	ILD(128Hz)		ILD(1336Hz)		Loundness		Sharpness		Tonality	
		coefficient	p value	coefficient	p value	coefficient	p value	coefficient	p value	coefficient	p value
Frisson	[monaural]	—	—	—	—	-0.39	0.30	0.77	0.015 *	-0.22	0.58
	[binaural]	0.93	0.000 ***	0.91	0.001 ***	-0.78	0.013 *	0.95	0.000 ***	-0.33	0.38
Pleasantness	[monaural]	—	—	—	—	0.12	0.77	-0.52	0.15	-0.07	0.85
	[binaural]	-0.76	0.018 *	-0.74	0.024 *	0.68	0.065	-0.76	0.019 *	0.22	0.57
